# Acute ischemic stroke and convexity subarachnoid hemorrhage in large vessel atherosclerotic stenosis: Case series and review of the literature

**DOI:** 10.1002/ccr3.5968

**Published:** 2022-06-19

**Authors:** Abeer Sabry Safan, Yahia Imam, Naveed Akhtar, Haya Al‐Taweel, Ayman Zakaria, Aiman Quateen, Ahmed Own, Saadat Kamran

**Affiliations:** ^1^ Department of Neurology Neurosciences Institute, Hamad Medical Corporation Doha Qatar; ^2^ Weill Cornell Medicine Doha Qatar; ^3^ Department of Neuroradiology Neurosciences Institute, Hamad Medical Corporation Doha Qatar

**Keywords:** case report, convexity subarachnoid hemorrhage, ICA stenosis, watershed infarct

## Abstract

Atraumatic convexity subarachnoid hemorrhage (cSAH) is a rare non‐aneurysmal SAH, commonly due to ipsilateral internal carotid artery (ICA) stenosis. It is unusual for the cSAH to occur contralaterally to the infarct. We report two cases of acute ischemic stroke associated with contralateral and ipsilateral cSAH that had different presentations.

## Graphical abstract

Due to diverse cSAH etiologies, physicians must have a low threshold to investigate possible large vessel stenosis in addition to the consideration of other common causes. If large vessel stenosis is diagnosed, then the patients' demographics and risk factors may play a role in risk stratification and management.

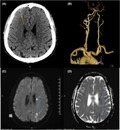

## 1 INTRODUCTION

Atraumatic convexity subarachnoid hemorrhage (cSAH) is an unusual non‐aneurysmal subarachnoid hemorrhage involving one or few adjacent sulci.^1^ Variable symptoms and etiologies have been described. Common etiologies include cerebral vasoconstriction syndrome in those 60 years old and below and cerebral amyloid angiopathy in those older than 60.^1^ Other etiologies such as cortical vein thrombosis and posterior reversible leukoencephalopathy (PRES) have also been described. Extracranial or intracranial artery stenosis such as internal carotid artery (ICA) or middle cerebral artery (MCA) stenosis with cSAH is rarely described.^8^ Furthermore, simultaneous acute ischemic infarct with cSAH has been reported infrequently, and most are ipsilateral to the infarct.^1^, ^2^ To our knowledge, there are two previous reported cases of acute ischemic stroke (AIS) with contralateral cSAH.^2^ We report two cases of severe ICA and M1 MCA stenosis‐associated acute infarction with contralateral and ipsilateral cSAH.

## 2 CASE PRESENTATION

### 2.1 Case 1

A fifty‐year‐old right‐handed man of South Asian race, with a past medical history of hypertension on lisinopril, presented to the emergency department (ED) with a 2‐day history of sudden onset right‐sided weakness, which subsequently improved with residual mild distal weakness. He denied any vertigo, diplopia, or other neurological deficits. There were no complaints of headache, witnessed loss of consciousness, or altered sensorium. He denied any history of trauma or similar symptoms in the past. Initial vitals were only significant for high blood pressure of 180/90 mmHg. Neurological examination showed a drift in the right upper and lower limb, with mild decreased pinprick sensation ipsilateral to the weakness sparing the face, scoring three on the National Institute of Stroke Scale (NIHSS).

**FIGURE 1:**
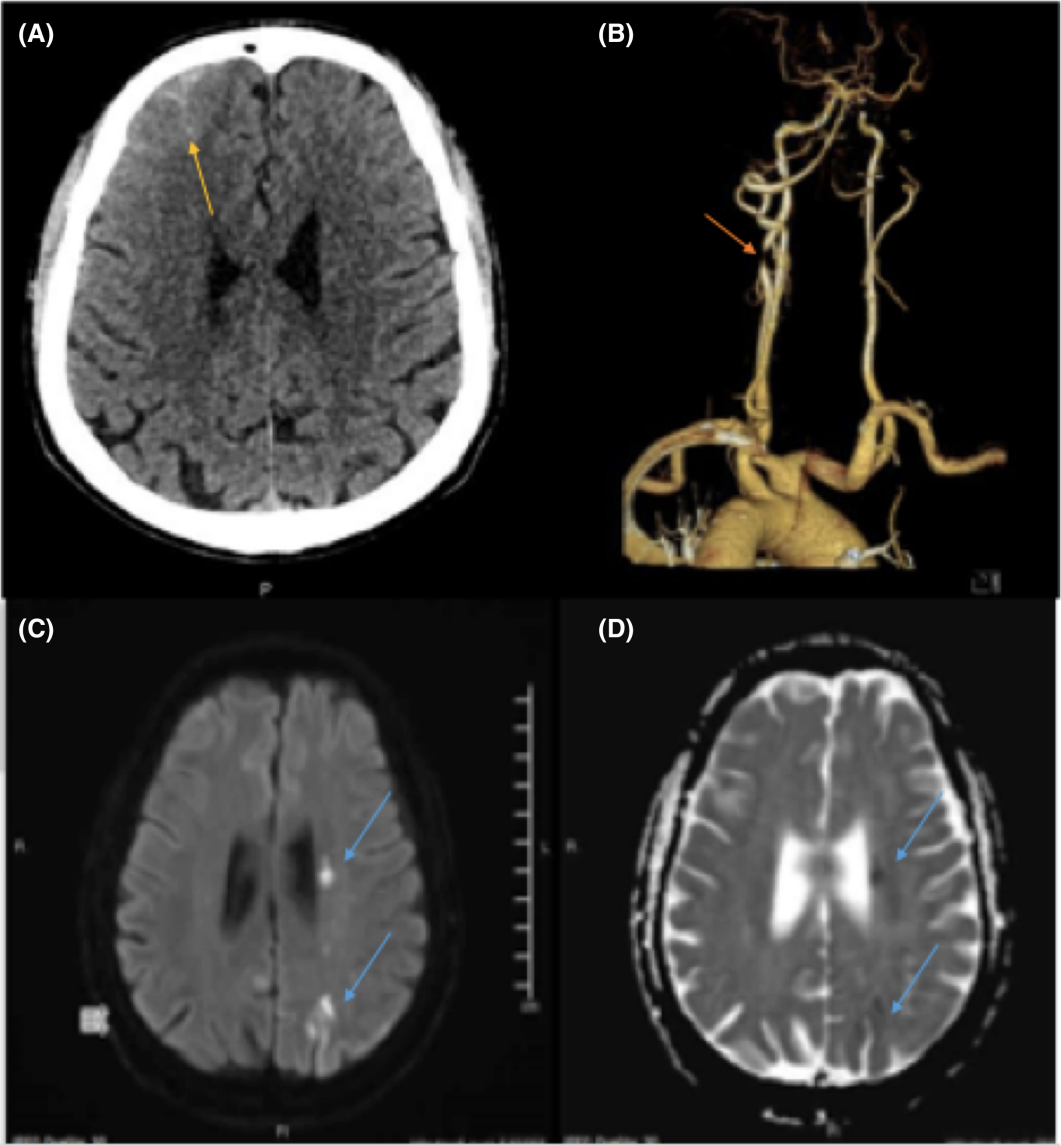
[A–D] Case one with Left ICA stenosis and contralateral cSAH. [A, B] None enhanced CT head: There is minimal subarachnoid hemorrhage along the right frontal sulci. CTA head and neck: There is severe short segment stenosis of the left proximal internal carotid artery. Extensive atheromatous calcification of the cavernous segments of the internal carotid arteries bilaterally. [C, D] MRI, diffusion‐weighted imaging (DWI) and apparent diffusion coefficient (ADC) multiple areas of watershed acute infarcts in the left hemisphere, with contralateral right frontal minimal subarachnoid hemorrhage

Initial laboratories showed normal complete blood count, renal function test and electrolytes, mildly elevated HbA1c of 6.6%, and cholesterol of 4.2 mmoL/dl with low‐density lipoprotein (LDL) of 2.7 units. Initial non‐contrast computed tomography (CT) of the head showed a convexity subarachnoid hemorrhage (cSAH) along the right frontal sulci (Figure 1A).

Computed tomography angiogram (CTA) showed no aneurysms and only high‐grade left ICA proximal severe stenosis (Figure 1). Subsequent magnetic resonance imaging (MRI) of the head showed multiple scattered increased intensity on diffusion‐weighted sequence in the left hemisphere (centrum semioval, corona radiata, body of caudate nucleus, anterior limb of internal capsule, and posterior parieto‐occipital cortical/subcortical areas) with corresponding low ADC map denoting areas of diffusion restriction, along with bright signal intensity on T2/FLAIR suggestive of acute ischemic infarcts (Figure 1D,E). Susceptibility‐weighted imaging (SWI) showed no cortical microbleeds and confirmed the presence of SAH blood.

Magnetic resonance venogram (MRV) showed no evidence of cortical or sinus venous thrombosis. Magnetic resonance angiogram (MRA) confirmed left ICA stenosis. The patient was started on aspirin 100 mg as a single antiplatelet with 40 mg atorvastatin daily as high‐intensity statin regimen. Trans‐thoracic echocardiography (TTE) showed mild concentric left ventricular hypertrophy, attesting to the long‐standing hypertension with average ejection fraction (52%), with no regional wall motion abnormality, and normal left atrial pressure.

The ICA stenosis was further corroborated with a carotid Doppler (CD) showing concentric intimal thickening of the left proximal ICA with extreme comprise in the luminal diameter and very high peak systolic velocity (PSV) of >230 cm/s and IC/CC PSV ratio >4.0 indicating more than 70% focal functional luminal stenosis. Hence, the diagnosis of left watershed infarct with contralateral cSAH in the setting of high‐grade ICA stenosis diagnosis was made. The vascular surgery team was consulted, and a carotid endarterectomy (CEA) was performed within 2 weeks from ictus.

The patient improved gradually and was discharged to follow up with a modified Rankin Score (mRS) of 1.

### 2.2 Case 2

A twenty‐nine‐year‐old right‐handed woman of southeast Asian race with no known past medical history presented to ED with a 3‐day history of severe holo‐cephalic headache that started after sneezing. The headache did not respond to paracetamol, and it was followed by nausea and vomited and new right‐sided numbness predominately the face and right upper limb a day later.

Her examination initially showed normal vitals and normal neurological examination except mildly impaired sensation in the right arm (NIHSS 1).

**FIGURE 2:**
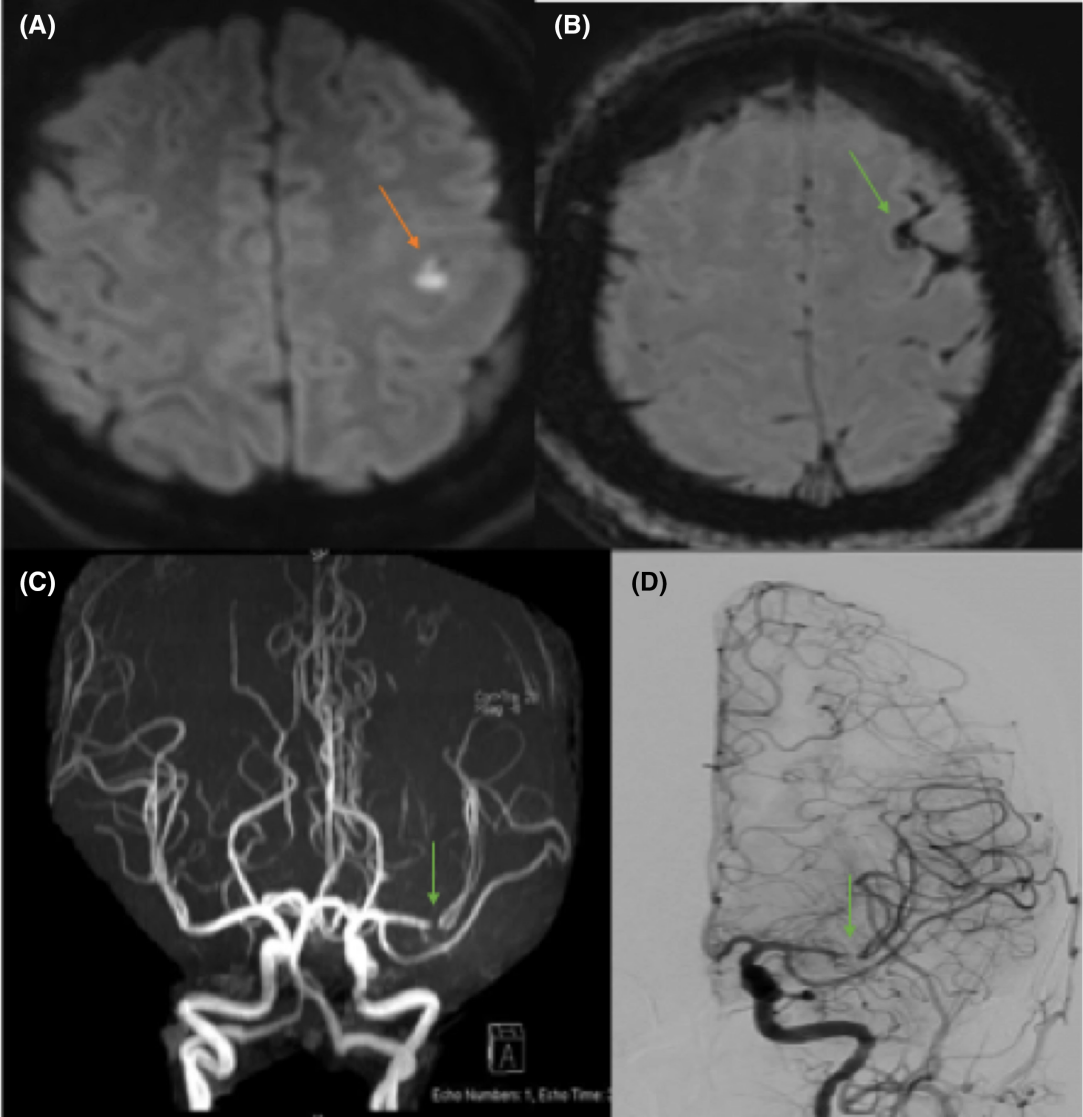
[A–D] Case two with left M1 stenosis an ipsilateral cSAH [A, B] There are effaced sulci on T1, with sulcal high FLAIR signal intensity, and blooming artifact seen within the left frontal sulci corresponding to the high density representing subarachnoid hemorrhage. [C] Cranial MRA: There is focal segment of severe stenosis seen at the M1/M2 of the left MCA, with faint visualization of the proximal M2 branches and paucity of distal M3 branches [D] Cerebral angiography: Tight stenosis of the distal Lt M1 segment. Sluggish flow in the Lt MCA branches, Lt transverse, and Lt sigmoid sinuses

Computed tomography (CT) of the head showed left cSAH (Figure 2A); CT angiogram did not show any aneurysms. Magnetic resonance imaging and angiography (MRI/MRA) of the head showed left frontoparietal acute ischemic changes with ipsilateral SAH in addition to M1/M2 severe stenosis (Figure 2B,C, and D) that raised the suspicion of dissection vs. reversible vasoconstriction syndrome given the presenting history. However, a conventional cerebral angiography showed only tight atherosclerotic stenosis of the distal M1 segment of the left MCA (Figure 2E) with no evidence of dissection or vasoconstriction.

Extensive blood workup, including connective tissue disease, drug, and amphetamine screening, homocysteine levels, antiphospholipid, and anticardiolipin antibodies, was unrevealing. Trans‐thoracic echocardiogram (TTE) was unremkarable. The patient was diagnosed with isolated intracranial atherosclerosis.

The patient was started on dual antiplatelets with aspirin 100 mg and clopidogrel 75 mg daily for 90 days. Her headache throughout her stay improved and resolved; a follow‐up MRI in 10 days showed significant interval regression ipsilateral cSAH and significant regression of previously described two focal left‐hemispheric subcortical ischemic changes. Hence, diagnosis of acute ischemic stroke and ipsilateral cSAH in presence of M1 segmental stenosis. One patient was discharged and was in good condition upon follow‐up with adherence to medical management.

## 3 DISCUSSION

Atraumatic convexity subarachnoid hemorrhage is a rare yet increasingly recognized subtype of subarachnoid hemorrhage. It is characterized by localized bleeding in one or few cortical sulci without the typical radiological findings of aneurysmal SAH, such as the extension into the interhemispheric fissures, ventricles, or basal cisterns.^1^ Pathophysiology of cSAH is not fully understood and is variable according to the underlying etiology, age, and population.^1^

The etiology of cSAH is variable and includes a spectrum of vascular and nonvascular pathophysiologies. Some are commonly recognized causes, such as cortical vein thrombosis, PRES, posterior reversible leukoencephalopathy syndrome (RCVS), ICA stenosis, cavernoma, cerebral vasculitis, and thrombocytopenia, while others can be related to anti‐coagulation and thrombophilia.^1^

A large retrospective study on 460 patients with SAH identified 29 patients fulfilling the criteria of atraumatic cSAH; 75% of those patients who were ≤60 years old presented with thunderclap headaches, with the most typical cause being RCVS, while those above the age of 60 years had variable transient sensory and motor symptoms with cerebral amyloid angiopathy being the most typical cause in this age group.^1^ Interestingly, unusual presentation in a small group of patients exhibiting altered sensorium and fever without evidence of infectious etiology was also reported.^3^

Acute ischemic infarct and cSAH have underlying correlating pathophysiology that is not fully understood. The development and evolution of the collateral circulation are likely central to the development of this condition.

As seen in atherosclerotic disease, progressive arterial occlusion allows for the development of robust collateral circulation in most cases. While the presence of the collateral circulation is a compensatory protective mechanism against cerebral ischemia, the pathophysiology and the clinical outcome may be more complicated.

On the one hand, two abnormal angiographic patterns were observed by Choi et al.; one where there is robust development of leptomeningeal collaterals causing a shift pattern of the angiographic ACA‐MCA border zone toward the MCA side as compared to the normal side and decreased MCA vessel size, with relatively normal maintenance of MCA flow. The other is the dilatation pattern, which appears to be related to poor development of the leptomeningeal collaterals, leading to dilatation of the cerebral vessels, suggesting a loss of an autoregulation response.^9^ The authors then conclude that “patients with the dilatation pattern tended to have more strokes and border zone infarcts than those with the shift pattern", indicating that the dilatation pattern may be an angiographic risk factor for symptomatic watershed ischemic stroke.^9^ (Diagram 1) Additionally, astereognosis is complex and includes arteriogenesis with the new vessels more prone to injury due to blood flow hemodynamics, altered shear stress, inflammation, activation of the endothelium, and remodeling. This makes them prone to bleeding, similar to what is proposed in moyamoya disease.^3^

The etiology of AIS co‐occurring with cSAH differs depending on the location, which the underlying pathophysiology can explain. Although the overall prevalence of such presentation is rare, AIS, most commonly, has severe ICA stenosis. cSAH, when it occurs in the context of AIS, is usually ipsilateral; however, contralateral occurrence such as our patient is rarely reported see (Table 1).

**TABLE 1:** Literature review of Acute ischemic stroke and cSAH

	Kumar^1^	Chandra^4^	Geraldes^10^	Nakajima^7^	Cho^9^	Larrosa^8^	Zhao^13^	Cao^2^	Introna^5^	Takamiya^12^	Sato T.^6^	Qin^11^
Year	2010	2011	2014	2014	2015	2016	2017	2019	2019	2019	2020	2020
Type of study	Retrospective review	Case report	Case Series	Retrospective study	Retrospective study	Case report	Retrospective study	Case series	Retrospective study	case series	Systematic review?	Case report
*N*	29	1	15	8 cSAH/ total 4953	15	1	14 cSAH/144 patients	2	4	2	24 (SAH), 5 cSAH	1
Mean age	58	70	65	71	57.5	78	62	62	64	44	50	33
Presenting signsand symptoms	75% severe headache, 54% transient focal neurological symptom (motor, sensory)	recurrent transient neurological deficits (aphasia, dysarthria, numbness)	86.6% focal neurological deficits 13.4% acute headache	headache, seizures, focal neurological deficits	53% focal neurological deficits (hemiplegia), 13% LOC, 13% headache, 6.6% Focal seizures, 6.6% dizziness	asymptomatic, 7‐year follow‐up presented with dysarthria and right facial paresis	57% headache, 57% focal neurological deficit	focal neurological deficit	headache, focal neurological deficits	headache	headache, focal neurological deficit	headache and left‐sided weakness
predominant cSAH location	51% ipsilateral frontal, 27.5% parietal 17% bihemispheric.	ipsilateral, central sulcus	ipsilateral, frontal	ipsilateral, 37.5% frontal, 37.5% parietal, 25% frontoparietal	ipsilateral, frontal	ipsilateral	ipsilateral parietal 71.4%, 64.3% frontal	contralateral parietal	ipsilateral, frontal	ipsilateral, frontotemporal	ipsilateral, frontal	ipsilateral frontal
Brain CT/MRI FLAIR DWI, T2	24% microbleeds (cortical and deep), 17.2% Superficial siderosis, 2.4% PRES. No ischemic infarcts	CTA: 80% ICA origin stenosis. MRI: no acute infarction.	33.3% significant ICA stenosis, 13.3 CAA, 13.3 RCVS, 6.6% CVST, 13.3, Dural fistula, 20% undetermined	Cases III and VII: acute ischemic infarct	73% had acute ipsilateral infarct, 27.2% ICA stenosis, 33.3% MCA stenosis, 13.3% ACA stenosis, 6.6 CAA	50% cICA stenosis, 7‐year follow‐up, 90% cICA stenosis with and ACA territory infarct	85.7% unilateral cSAH, 14.2 bihemispheric cSAH 28.5% scattered acute subcortical infarcts (ipsilateral)	Case I: left MCA infarction case II: right MCA infarction	MRI: Case 1: subcortical ischemic infarct (lenticular nucleus), case 2: watershed/border zone infarct, case 3: cortical frontal, case 4: semioval center infarct	Case I: left frontal watershed infarct case II:	Cases I‐IV: MRI: ipsilateral MCA infarct. case V:	Right MCA infarction,
Cerebral Angio / DSA	14% arterial narrowing (ACA, MCA, PCA), 10.3% arterial dissection (cerebral, ICA)	cerebral angiography, no RCVS, vascular malformation, or vasculitis.	26.6% Ipsilateral ICA stenosis, 13.3% multiple intracranial stenosis (RCVS) + cSAH	cases I, IV‐VIII: angiography shows severe stenosis of major arteries (50% extracranial ICA, 12.5 MCA, 12.5 bilateral VA)	26.6% acute infarction, high‐grade extracranial stenosis and ipsilateral cSAH, 13% High‐grade intracranial stenosis with cSAH no acute infarction	left high‐grade cICA stenosis	71.4% has high‐grade^a^ stenosis/occlusion of ICA, MCA, PCA, 28.5% CVST, vascular malformation, CAA, undetermined	case I: occlusion of the left ICA and compensatory flow from the right ICA via the anterior communicatingartery case II: right ICA occlusion compensatoryflow from the ipsilateral anterior cerebral artery via the leptomeningeal artery	case II: MRA showed intracranial stenosis MCA, PCA. case I, III: DSA unremarkable. case IV: ICA stenosis	case I: (DSA) left ICA and(MCA) stenosis, pial anastomosis fromthe left PCA case II: (DSA) right ICA stenosis, pial anastomosis from the right ACA, PCA	cases: I‐‐IV: angiography: MCA stenosis. DSA: no vascular malformation or RCVS.	DSA: high‐grade, MCA M1‐segment stenosis
Carotid Doppler/TCD Other examinations	EEG. no epileptiform discharges (in patient with transient neurological symptom/sensory march)	continuous EEG: No epileptiform waves,	ipsilateral atheromatous ICA stenosis	SPECT: 99 m Tc in case I: decreased CBF ipsilateral cerebral hemisphere	27.2% of acute infarction on (DSC)—hyperperfusion state of infected area	TCD: cerebral vasoreactivity in the left anterior territory	NA	Case I: case II: TTE: rheumatic heart disease with aortic stenosis	Case1: TCD‐TTE, PFO. case 2: intracranial stenosis MCA, PCA. case I, III: DSA unremarkable	123I‐IMP single PET, decreased flow in ipsilateral cerebral hemisphere	CTV: one has cortical CVST	hyperhomocysteinemia,
Etiology	RCVS, HELLP/PRES, FMD, IE (mycotic aneurysm), amyloid angiitis	atherosclerosis	ICA stenosis, CAA, CVST, RCVS, undetermined	atherosclerosis, CAA,	large vessel disease (ICA, McA, ACA stenosis) CAA, undetermined	cICA progressive stenosis	atheromatous disease (high‐grade stenosis), CAA, CVST	ICA occlusion (Large vessel disease)	Case I: PFO, case II‐IV: atheromatous disease, case III: ESUS	Large vessel disease (Atherosclerosis)	atherosclerosis, venous infarction	MCA M1 segment stenosis
Treatment (according to etiology)	NA	emergent ipsilateral CEA	antiplatelet and Statin, endarterectomy, anti‐coagulation	antiplatelets	single antiplatelet and statin, endarterectomy	single antiplatelet and statin, endarterectomy	single antiplatelet, anti‐coagulation.	Case I: single antiplatelet and Statin case II: anti‐coagulation (warfarin)	single antiplatelet, closure of PFO, carotid revascularization.	Case i: left superficial temporal artery‐MCA bypass. case II: antiplatelet	Antiplatelet	Shuxuetong injection to improve cerebralcirulaitonadn metabolism, edaravone to eliminate free radicals, Folic acid, B6, B12 to reduce homocysteine. Single Antiplatelet and Statin

Abbreviations: ACA arterioiranterior cereberalcerebral artery; CAA, Cerebrlacerebral amyloid angiopathy angiopathy; CEA, cCarotid endarterectomy; cICA, cervical internal carotid artery; CVST, cortical venous sinus thrombosis; DSC, dynamic susceptibility contrast perfusion study; ESUS, eEmbolic stroke of undetermined stroke; FMD, fibromuscular dysplasia; IE infective endocarditis; MCA, Mmiddle cerebral artery; NA, not available; PCA, posterioirposterior cerebral artery; PECT, positoeronpositron emission computed tomography; PRES, posterioirposterior reversible encephaloapthyencephalopathy syndrome; VA, Vertebral artery.

^a^ High‐grade stenosis >70%.

Ipsilateral cSAH is explained by the formation of compensatory collaterals' susceptibility to rupture and bleed at any time.^4^ Contralateral cSAH is much rarer than ipsilateral and may have slightly different underlying pathophysiology. It is well documented that chronic ICA stenosis causes several compensatory mechanisms to maintain blood flow to the brain.^5^ One compensatory mechanism is the contralateral dilatation of vasculature in order to preserve blood flow as explained previously. However, this compensatory mechanism can be harmful if the vessels expand beyond physiological limits leading to increased vessel fragility and rupture.^4^, ^5^

In addition, another studied compensatory mechanism is the establishment of contralateral collateral circulation to maintain blood and perfuse the areas of the brain supplied by the occluded vessels.^5^ Nonetheless, similar to dilated vessels, collateral vessels are usually fragile in structure, making them highly susceptible to rupture, causing hemorrhage in the form of cSAH.^4^, ^5^

A study involving 384 patients with symptomatic ischemic stroke aimed to measure the incidence of cSAH with AIS. Six of the 384, 382 had arterial AIS, and two had venous AIS. Of the arterial AIS, only two patients (0.5%) are identified with cSAH within 4.5 h of presentation.^6^ Within 6 days of presentation, another two patients (0.5%) were identified.^6^ Another study measuring the incidence of cSAH with concomitant AIS found that over half of the patients with cSAH concurrently had an occlusion of a major artery.^7^ These findings suggest that the primary, most common etiology behind cSAH could be an underlying significant arterial occlusion, mainly involving the extracranial ICA.

Carotid revascularization is well established for high‐grade ipsilateral symptomatic carotid disease, and this mainstay treatment has been shown to significantly decrease the average annual stroke rate and improve patients' outcomes and prognosis.^8^ Hence, it is possible to provide the same treatment and medical therapy to patients with concomitant cSAH.^8^ Medical treatment can be individualized to the patient's condition; however, it usually includes antiplatelet agents (aspirin or clopidogrel), statins, and antihypertensive medications, along lifestyle modifications.^8^ However, the presence of cSAH even in the absence of stroke may be considered a marker for altered hemodynamic and may tip the scale toward carotid revascularization.^12^ At the same time, the best medical therapy (90 days of dual antiplatelet) based on the SAMPRIS trial remains the standard for patients with symptomatic severe intracranial stenosis with cSAH.^14^ However, it is unknown whether antiplatelet therapy or more interventional therapy dictated by the presence of cSAH is beneficial; hence, large‐scale evidence‐based medicine research is warranted.

## 4 CONCLUSION

cSAH could coincide with acute ischemic stroke. It could be ipsilateral or contralateral. It is essential to search for large artery occlusion actively, whether ipsilateral or contralateral, and the presence of significant stenosis in the presence of cSAH might arguably tip the scale toward more aggressive intervention.

## AUTHOR CONTRIBUTIONS

AS, HA, YI, and NA wrote the initial draft of the manuscript. YI, NA, SK, AZ, AQ, and AO conceptualized and supervised the study. HA, AS, YI, NA, and SK contributed to the medical management of the case. AS, HA, YI, NA, SK, AZ, and AQ revised the manuscript critically and contributed to the literature review. All authors have read and approved the manuscript.

## CONFLICT OF INTEREST

The authors have no conflict of interest to disclose.

## ETHICAL APPROVAL

This case report was approved by the Hamad Medical Corporation's Medical Research Center (Protocol number: MRC MRC‐04‐21‐512).

## CONSENT

Written informed consent was obtained from the patient for publication for this case report and any accompanying images. A copy of the written consent form is available for review by the Editor of this journal.

## Data Availability

The datasets used and/or analyzed during the current study are available from the corresponding author on request.

## APPENDIX 1 Atuhor Conritbutions

**Table:**

Name	Location	Contribution
Abeer Sabry Safan	Department of Neurology, Neurosciences Institute, Hamad Medical Corporation, Doha, Qatar	Writing the initial draft of the manuscript, medical management of the case, revising the manuscript critically, and literature review
Yahia Imam	Department of Neurology, Neurosciences Institute, Hamad Medical Corporation, Doha, Qatar	Conceptualization and supervision, medical management, revising the manuscript critically, and literature review
Naveed Akhtar	Department of Neurology, Neurosciences Institute, Hamad Medical Corporation, Doha, Qatar.	Conceptualization and supervision, medical management, revising the manuscript critically, and literature review.
Haya Al‐Taweel	Weill Cornell Medicine of Cornell University (WCMCQ), Doha, Qatar	Writing the initial draft of the manuscript, medical management of the case, revising the manuscript critically, and literature review
Ayman Zakaria	Department of Neuroradiology, Neurosciences Institute, Hamad Medical Corporation, Doha, Qatar	Conceptualization and supervision, revising the manuscript
Aiman Quateen	Department of Neuroradiology, Neurosciences Institute, Hamad Medical Corporation, Doha, Qatar	Conceptualization and supervision, revising the manuscript
Saadat Kamran	Department of Neurology, Neurosciences Institute, Hamad Medical Corporation, Doha, Qatar	Conceptualization and supervision, medical management, revising the manuscript critically, and literature review
Ahmed Own	Department of Neuroradiology, Neurosciences Institute, Hamad Medical Corporation, Doha, Qatar	Conceptualization and supervision, revising the manuscript
